# Bolometric photodetection using plasmon-assisted resistivity change in vanadium dioxide

**DOI:** 10.1038/s41598-018-30944-2

**Published:** 2018-08-24

**Authors:** Hironobu Takeya, James Frame, Takuo Tanaka, Yoshiro Urade, Xu Fang, Wakana Kubo

**Affiliations:** 1 Division of Advanced Electrical and Electronics Engineering, Tokyo University of Agriculture and Technology, 2-24-16 Naka-cho, Koganei-shi, Tokyo 184-8588 Japan; 2 School of Electronics and Computer Science, University of Southampton, Southampton, SO17 1BJ UK; 3 Metamaterials Laboratory, RIKEN Cluster for Pioneering Research, 2-1, Hirosawa, Wako, Saitama 351-0198 Japan; 4 Department of Physics, Faculty of Science, Gakushuin University, 1-5-1 Mejiro, Toshima-ku, Tokyo 171-8588 Japan; 5 Innovative Photon Manipulation Research Team, RIKEN Center for Advanced Photonics, 2-1 Hirosawa, Wako, Saitama 351-0198 Japan; 6 Department of Chemical Science and Engineering Major in Chemical Science and Engineering, School of Materials and Chemical Technology, Tokyo Institute of Technology, 4259 Nagatsuta-cho, Midori-ku, Yokohama, Kanagawa 226-8503 Japan; 7 Department of Electronic Science and Engineering, Kyoto University, Kyoto, 615-8510 Japan

## Abstract

Vanadium oxide is a key sensing material for bolometric photodetection, thanks to its strong temperature dependence of resistivity close to room temperature. Here we demonstrate the photodetection of a stoichiometric vanadium dioxide thin film integrated with silver nanorods. The nanorods convert light into heat, consequently suppressing the resistivity of vanadium dioxide via localised surface plasmon resonance. Incorporation of this thermo-plasmonic effect into bolometric photodetection allows for wavelength and polarisation sensitivity. This work opens the path to a broad family of photodetection functionalities for vanadium dioxide-based microbolometers.

## Introduction

Bolometric photodetection utilises the dependence of material resistivity on temperature to detect light. Due to its unique properties such as extremely broadband response and high sensitivity, it is chosen for many applications including uncooled infrared imaging and single photon detection^1–3^. At present, vanadium oxide is the most widely used sensing material in microbolometers^4^. Several unique properties of the material have led to its market dominance, including strong light absorption and an orders-of-magnitude change in resistivity (i.e. a high temperature coefficient of resistance) close to room temperature^5^.

Among the various phases of vanadium oxide, stoichiometric vanadium dioxide (VO_2_) is frequently chosen for proof-of-principle demonstrations, as its simple composition allows for high reproducibility of experimental results^6–8^. An emerging research topic of VO_2_ is its interplay with surface plasmons^9–15^. Previous work has found that plasmonic resonances can facilitate the phase change of VO_2_ and the phase change can in turn modulate plasmonic resonances^16^. Most of this research has been conducted in the context of tuneable plasmonic devices and reconfigurable metamaterials. In contrast, this work focuses on utilising plasmons to introduce new functionalities (here wavelength and polarisation sensitivity) to bolometric photodetection. As far as we know, this is the first report on the electrical detection of light via plasmonic resonances in VO_2_. Compared to plasmon-assisted photodetection on other material platforms^17^, our choice of VO_2_ lends our work immediate relevance to practical applications. In particular, although VO_2_-based photodetection lacks several functionalities available in 2D materials (e.g. ultrafast photodetection in graphene^18^), its mature technology platform is ideal for testing the integration of sensing materials and novel plasmonic nanostructures.

**Figure 1:**
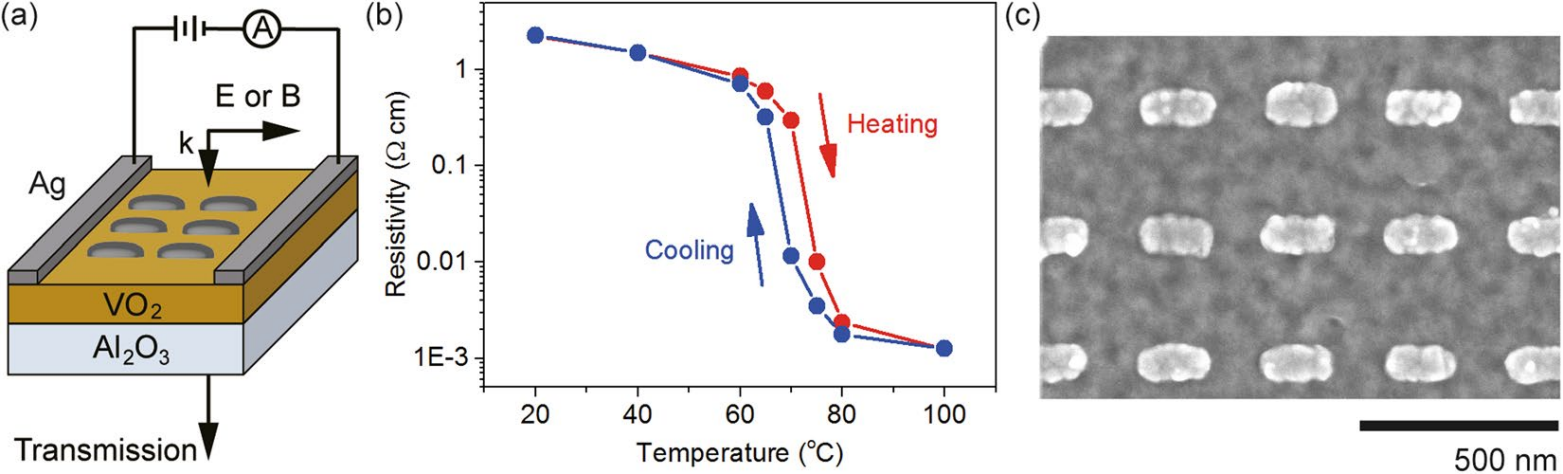
VO_2_ bolometer integrated with silver (Ag) nanorods. (**a**) Schematic of the sample and its measurement configuration. The sample consists of two Ag electrodes and an array of Ag nanorods on top of a VO_2_ film, which is supported by a bulk Al_2_O_3_ substrate. The nanorods are illuminated by light linearly polarised along either their short or long axis. The temperature is controlled and monitored using a Peltier element and a temperature sensor (not depicted). (**b**) Resistivity hysteresis loop measured between the two electrodes before the fabrication of nanorods. (**c**) SEM images of a section of the nanorod array.

Figure 1(a) schematically illustrates the sample and its measurement configuration. The sample consisted of a thin layer of VO_2_ prepared by magnetron sputtering on a bulk sapphire (Al_2_O_3_) substrate. Two rectangular silver (Ag) electrodes were subsequently grown on the sample surface using thermal evaporation. The high quality of the VO_2_ film was verified by measuring the dc resistivity between the electrodes in a thermal cycle (Fig. 1(b)). The resistivity changed by over three orders of magnitude between 20 °C and 100 °C, and showed a phase transition temperature, defined as the centre of the resistivity hysteresis loop, of ~68 °C.

A periodic array of Ag nanorods was subsequently patterned between the two electrodes following a standard electron beam lithography and lift-off process. The array was 1.8 mm × 1.8 mm in size and was located at the middle of the two electrodes. The nanorods had a thickness of 40 nm, averaged planar dimensions of 95 nm × 180 nm and a periodicity of 300 nm × 300 nm (Fig. 1(c)). Ag was chosen as the nanorod material for these two reasons. (1) Ag has extremely low resistivity (1.6 × 10^−6^ Ω·cm at 20 °C) compared to VO_2_ (measured as 2.3 Ω·cm at 20 °C), making a small change in the VO_2_ relatively easy to detect. (2) Ag supports strong localised surface plasmon resonance (LSPR) within the entire wavelength range of interest, thus enabling investigation of the thermo-plasmonic effect.

The VO_2_-nanorod sample obtained after nanofabrication was characterised using two home-built systems. System A consisted of an optical microscope (Olympus BX51) and a fibre-coupled spectrometer (Ocean Optics HR4000) for measuring the transmission. Meanwhile, System B consisted of a Xenon lamp (Hamamatsu Photonics E7536) and a monochromator (Shimadzu SPG-120S) for measuring the dc resistivity under light illumination. In both systems, the nanorods were illuminated at normal incidence, with the light linearly polarised along either their long or short axis. The light was focused to the centre of the array, and the focal spot was slightly smaller than the array. The temperature of the sample was maintained at 66 °C, a value slightly below the phase transition temperature, for transition-edge detection. This temperature control was achieved by using a Peltier module, which was attached to the bottom of the Al_2_O_3_ substrate and a temperature sensor, which was placed at the surface of the VO_2_ film close to the nanorod array.

**Figure 2:**
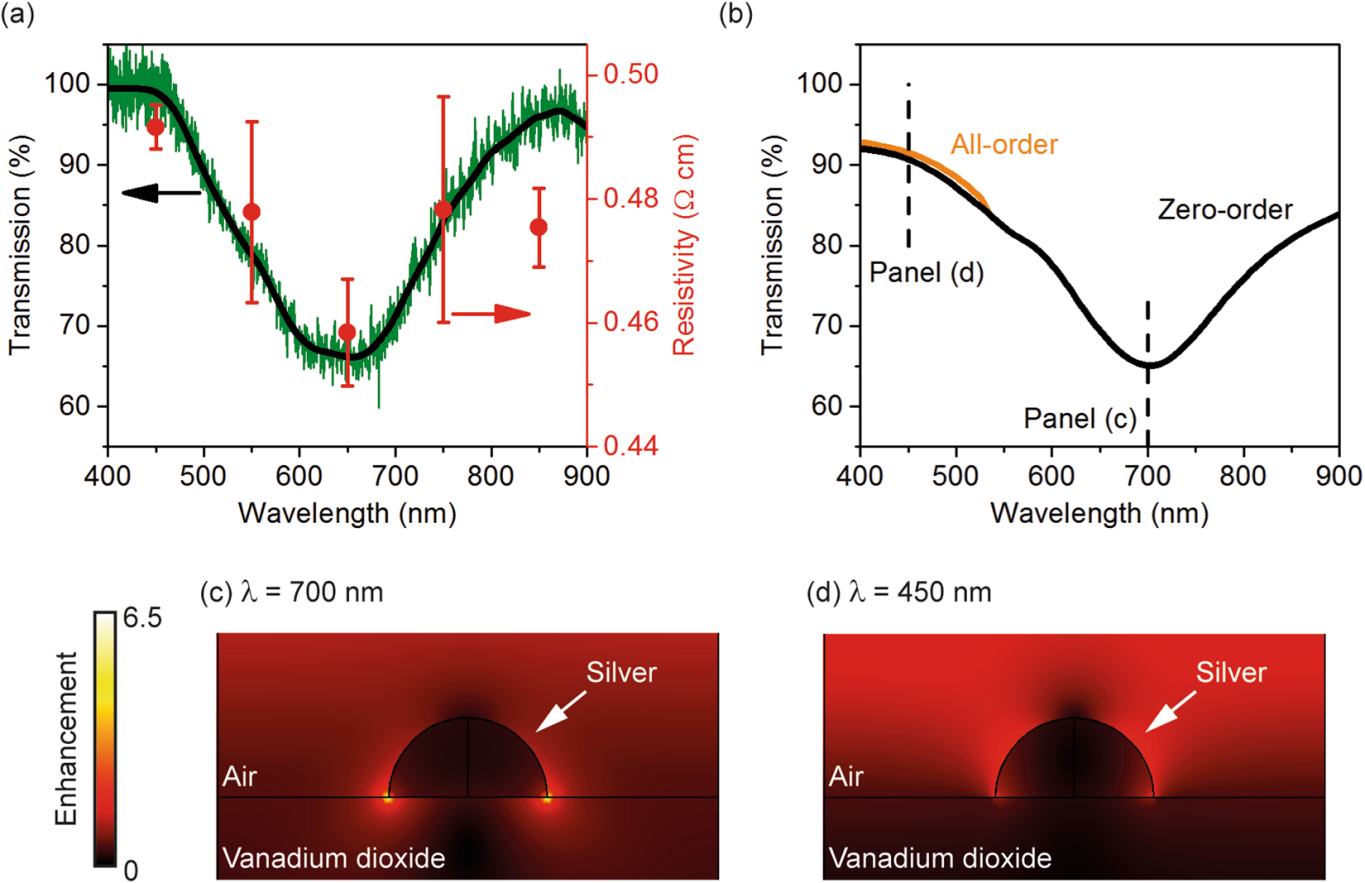
Optical and optoelectronic properties of the sample. (**a**) Experimentally measured optical transmission and wavelength dependent resistivity of the sample. The incident light is polarised along the short axis of the nanorods. The green and the black line are the measured and smoothed spectra, respectively. (**b**) Numerically simulated transmission of the sample. High-order forward diffraction is observed at wavelengths below 530 nm. (**c**,**d**) Electric field distribution at a 2D plane bisecting a nanorod along the short axis at a wavelength of **(c)** 700 nm and **(d)** 450 nm. The enhancement factor is with respect to the incident field.

Figure 2(a) shows the transmission spectrum of the sample measured using System A, where the intensity of incident light was kept at a very low level to minimise any thermal effect. The incident light was polarised along the short axis of the nanorods. A plain area of the same VO_2_ film was used as reference. The spectrum shows a strong and broad feature at 650 nm corresponding to a ~30% drop in transmission compared to transmission at 900 nm and 400 nm. Figure 2(a) also shows the resistivity measured using System B, where the sample was illuminated by light narrow in spectral width (~ 46 nm in full-width at half-maximum) and high in intensity (~10 mW·cm^−2^). The central wavelength of the incident light was tuned from 450 nm to 850 nm at a step of 100 nm, with the resistivity between the two electrodes measured at each wavelength. As VO_2_ has very small thermal conductivity (6 W·m^−1^·K^−1^)^19^, the nanorod array could drift in temperature with time under light illumination. To minimise such influence on the resistivity measurement, the whole wavelength range was swept four times; Fig. 2(a) shows the average and standard error of these four measurements.

The resistivity in Fig. 2(a) is wavelength dependent, and it roughly correlates with the transmission. Both values are smallest at 650 nm and largest at ~450 nm. To interpret this correlation, the electromagnetic behaviour of the sample is numerically simulated using a 3D finite-element solver (COMSOL Multiphysics). The nanorod is treated as a hemi-spherocylinder with dimensions taken from experimental values. The wavelength dependent permittivity of Ag and Al_2_O_3_ is taken from refs^20,21^, respectively. The permittivity of VO_2_, which depends on both the wavelength and metallic volume fraction (i.e. the filling factor of metallic phase in a dielectric matrix)^22^, is taken from ref.^23^. The volume fraction is set as 0.8 for the best fitting to experiment, and its influence on the transmission spectrum is shown in Fig. S2.

Figure 2(b) shows the numerically simulated transmission spectrum, which reproduces the experimental result reasonably well. High-order diffraction in the forward direction is observed for wavelengths below 530 nm. The zero-order spectrum corresponds to the measured spectrum due to the large distance between the sample and detector in the experiment. Compared to experiment, the simulated spectrum shows a redshift of ~50 nm, which is attributed to the difference in nanorod dimensions and material permittivity between the experiment and simulation (see Supplementary Information for details^24,25^). Figures 2(c,d) show the electromagnetic near-field distribution at respectively 700 nm, the wavelength with the smallest transmission in simulation, and 450 nm, the shortest wavelength for resistivity measurement. At 700 nm, the nanorod shows strong and highly confined enhancement of electromagnetic field. The electric field increases by a factor of ~6.5 compared to the incident light; this level of enhancement is only observed at the two sharp edges of the nanorod. These characteristics indicate that the broad and strong transmission feature is induced by the LSPR of the nanorods. The LSPR is significantly less pronounced at 450 nm as both the enhancement and confinement are much weaker (Fig. 2(d)). Figures 2(b,c) show that transmission amplitude directly reflects LSPR strength, with low transmission corresponding to strong LSPR.

We consequently attribute the correlation between transmission and resistivity in Fig. 2(a) to the thermo-plasmonic effect. Plasmonic nanostructures are well-known for their high efficiency in converting electromagnetic energy to thermal energy. Although considered as a key limiting factor for many applications, this effect has found several applications including reconfigurable metamaterials, heat-assisted magnetic recording, photothermal therapy, plasmon-assisted chemistry and plasmon-enhanced optical trapping^26–29^. For our sample, conversion of light to heat is most efficient at the LSPR resonance wavelength (650 nm in experiment and 700 in simulation). The nanorod array is at a slightly higher temperature than the temperature sensor nearby^30^, and the VO_2_ beneath the array is slightly suppressed in resistivity. For wavelengths far away from the resonance wavelength, the heating effect induced by the array is smaller, and the suppression in the resistivity is also smaller.

**Figure 3:**
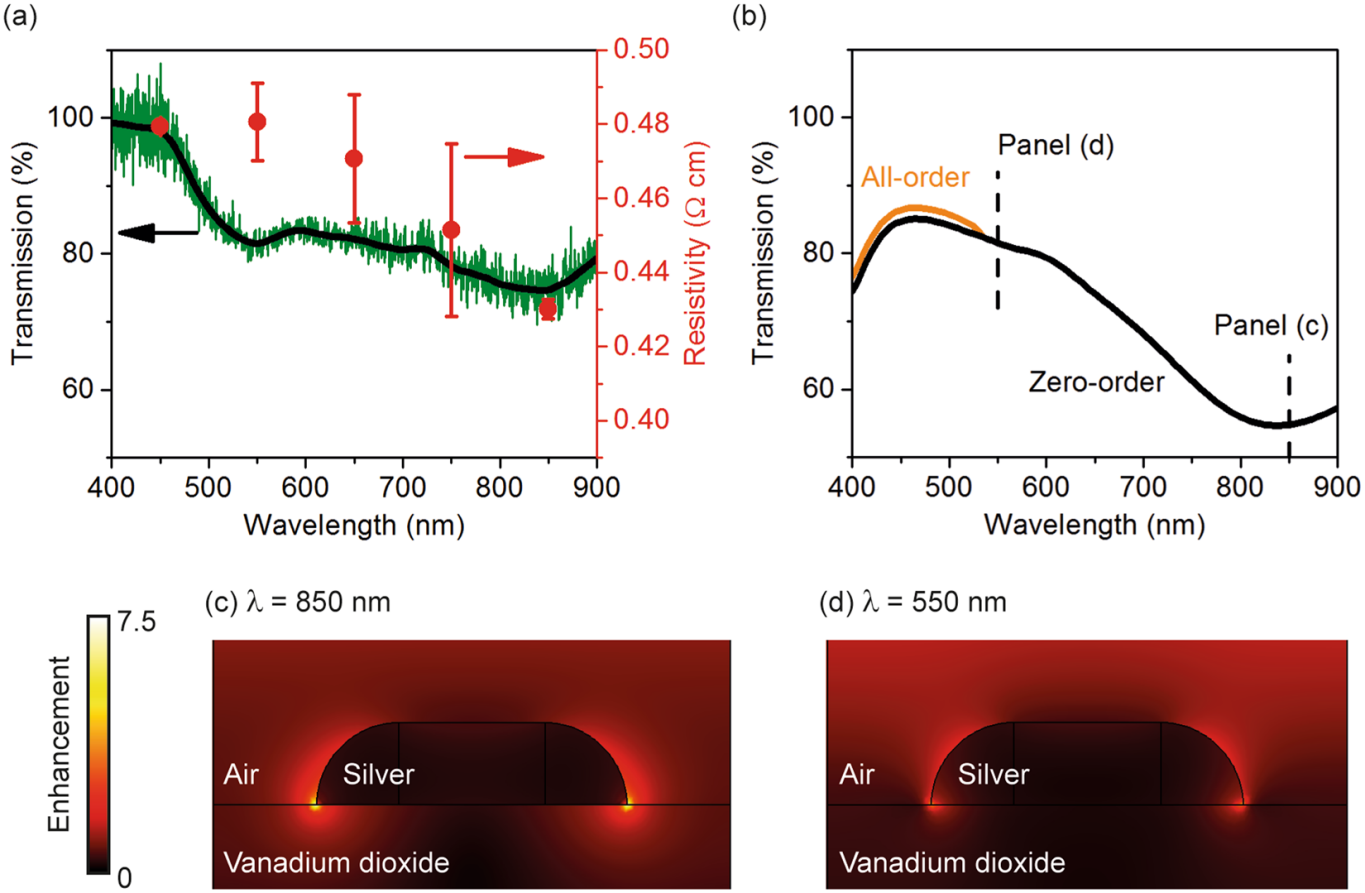
Sample properties under long-axis light excitation. (**a**) Experimental optical transmission and electrical resistivity. The green and the black line are the measured and smoothed spectra, respectively. The incident light is polarised along the long axis of the nanorods. (**b**) Numerically simulated transmission of the sample. (**c**,**d**) Electric field distribution at a 2D plane bisecting a nanorod along the long axis at a wavelength of **(c)** 850 nm and **(d)** 550 nm.

In addition to the wavelength dependence shown in Fig. 2, we further explored the polarisation dependence of the sample. Figure 3 shows corresponding results at the orthogonal light polarisation, i.e. the incident light is polarised along the long axis of the nanorods. The optical transmission shows two pronounced features at ~550 nm and ~850 nm, while the resistivity only traces the feature at ~850 nm (Fig. 3(a)). This difference originates from the different nature of the two spectral features: only the one at ~850 nm is induced by LSPR. This is supported by the near-field distribution in Fig. 3(c,d), where strong LSPR is only visible at 850 nm. The spectral feature at ~550 nm in Fig. 3(a), although not faithfully reproduced in simulation in Fig. 3(b), becomes more pronounced at smaller metallic volume fractions (Fig. S2). It is consequently attributed to the interplay between high-order diffraction and multiple interference inside the VO_2_ layer, as both phenomena depend on the permittivity of VO_2_, which in turn depends on the volume fraction. As this feature is not related to any near-field enhancement, no obvious suppression of resistivity is observed at this wavelength.

The difference between Figs 2 and 3 clearly shows the influence of plasmonic resonance on photodetection. The wavelength dependent resistivity in both figures, however, shows a relatively large standard error. Although a detailed discussion is beyond the scope of this proof-of-principle demonstration, we have noticed in ongoing experiments that covering the Ag nanorods with a thin layer of magnesium fluoride may significantly reduce the standard error. The cover layer is expected to protect the nanorods from tarnishing, as well as to stabilise the temperature distribution at the edges of the nanorods by reducing the influence of air convection. Rigorous experimental investigation and theoretical analysis of the optical, thermal and electrical behaviours of the device at the nanoscale will lead to higher device sensitivity.

To conclude, we have demonstrated bolometric photodetection using plasmon-assisted resistivity change in VO_2_. Ag plasmonic nanostructures grown on top of the VO_2_ film increase heat generation via the excitation of LSPR and consequently suppress its resistivity. This change in resistivity depends on the strength of the LSPR, which can be tuned by controlling the wavelength and polarisation of incident light. A rough and simple correlation between transmission and resistivity is observed for spectral features induced by LSPR. The nanorod array introduces a certain degree of wavelength and polarisation sensitivity to bolometric photodetection. As plasmonic nanostructures can facilitate advanced control over light, this work can lead to the development of multi-functional, VO_2_-based plasmonic microbolometers.

## Methods

### Sample fabrication

The sample consisted of a thin layer of VO_2_ (250 nm thick) on top of a bulk sapphire (Al_2_O_3_, ~1 mm thick) substrate. The VO_2_ layer was fabricated via reactive magnetron sputtering of vanadium under continuous flow of argon and oxygen gases (temperature = 450 °C, RF power = 200 W, Ar flow rate = 130 sccm, O_2_ flow rate = 3 sccm, total gas pressure = 0.5 Pa, and total sputtering time ≈ 2 hours). Two rectangular silver (Ag) electrodes were fabricated on the sample surface by thermal evaporation. These electrodes were 100 nm in thickness, 7.5 mm in length, and separated by 2.0 mm in between. A periodic array of Ag nanorods was patterned between the two electrodes following a standard electron beam lithography (JEOL JBX-6300FS) and lift-off process.

## Electronic supplementary material

supplementry information

## Data Availability

The data from this paper can be obtained from the University of Southampton ePrints research repository: 10.5258/SOTON/D0615.
