# Supplementary material for: Bolometric photodetection using plasmon-assisted resistivity change in vanadium dioxide
Source: Sci Rep. 2018 Aug 24;8:12764. doi: 10.1038/s41598-018-30944-2 (PMC6109045; doi:10.1038/s41598-018-30944-2)
Supplement: Supplementary file 1 — supplementry information [file 41598_2018_30944_MOESM1_ESM.pdf]

Bolometric photodetection using plasmon-assisted resistivity change in vanadium dioxide:

Supplementary Information

Hironobu Takeya,[1] *James Frame,*[2] *Takuo Tanaka,*[3,4,5,6] *Yoshiro Urade,*[7] *Xu Fang,*[2*] *and Wakana Kubo*[1,3*]

[1]Division of Advanced Electrical and Electronics Engineering, Tokyo University of Agriculture and Technology, 2-24-16 Naka-cho, Koganei-shi, Tokyo, 184-8588, Japan

[2]School of Electronics and Computer Science, University of Southampton, Southampton SO17 1BJ, UK

[3]Metamaterials Laboratory, RIKEN, 2-1, Hirosawa, Wako, Saitama 351-0198, Japan

[4]Department of Physics, Faculty of Science, Gakushuin University, 1-5-1 Mejiro, Toshima-ku, Tokyo 171-8588, Japan

[5]Innovative photon manipulation research team, RIKEN Center for Advanced Photonics, 2-1 Hirosawa, Wako, Saitama 351-0198, Japan

[6]Department of Chemical Science and Engineering Major in Chemical Science and Engineering, School of Materials and Chemical Technology, Tokyo Institute of Technology, 4259 Nagatsuta-cho, Midori-ku, Yokohama, Kanagawa 226-8503, Japan

[7]Department of Electronic Science and Engineering, Kyoto University, Kyoto 615-8510, Japan

*x.fang@soton.ac.uk
*w-kubo@cc.tuat.ac.jp

In comparison to many other materials (e.g. Au and Si_3N_4)[24,25], Ag and VO_2 have unique material properties that make numerical simulation for plasmonic metamaterials very challenging. Ag nanoparticles tarnish rapidly once they are exposed to air. The tarnish layer is known to increase the damping of plasmonic resonance, but the magnitude of such increase is difficult to identify. On the other hand, VO_2 shows complicated changes in crystalline structure during phase transition[22], which makes its permittivity highly temperature sensitive. Figures S1 and S2 numerically demonstrate the possible influence of these unique material properties on our sample. In Fig. S1, the damping constant of Ag is multiplied by a factor of 2 and 3. In Fig. S2, the permittivity of VO_2 changes between the two pure phases following the standard Maxwell Garnet effective medium model.

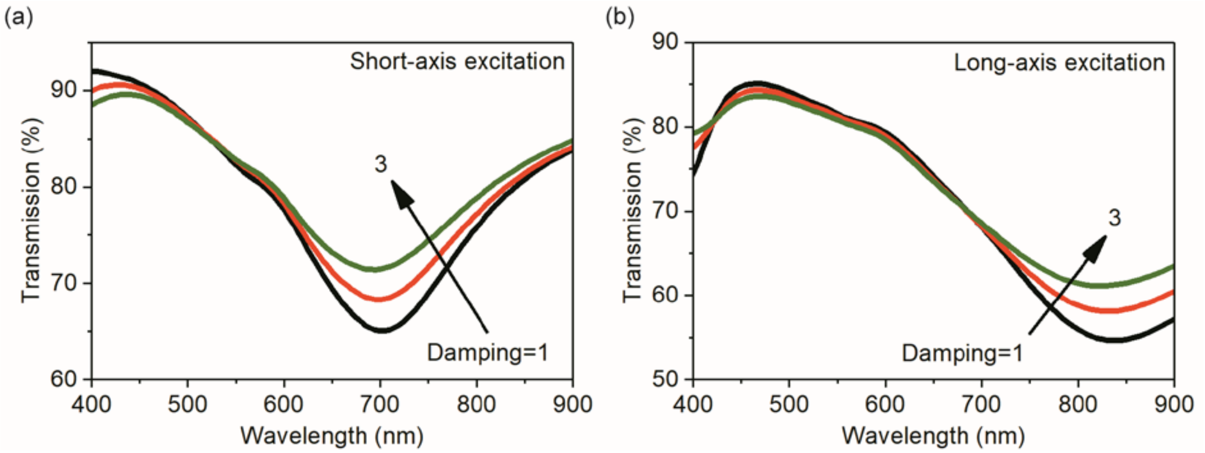

Figure S1. Influence of the Drude damping of Ag on the zero-order transmission. For most simulated results presented in this manuscript, the damping constant of Ag is the same as in Ref. 20. Here this constant is multiplied by a factor of 2 (red lines) and 3 (green lines). The plasmonic resonances at 700 nm and 850 nm are weaker with increased damping.

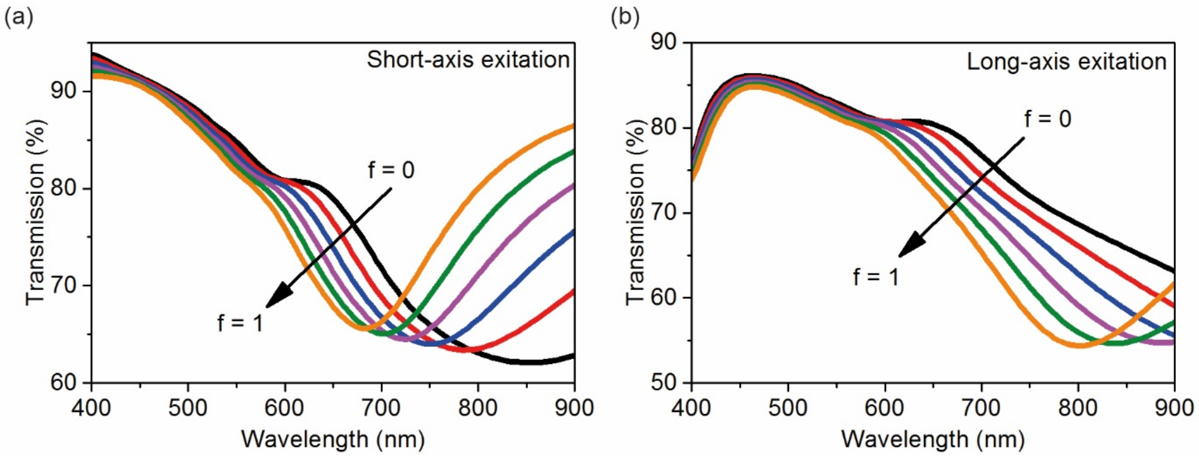

Figure S2. Influence of metallic volume fraction *f*, the relative volume of metallic phase inside a dielectric matrix, on the zero-order transmission. In this series of numerical simulation, *f* changes from 0 (the low-temperature, pure dielectric state) to 1 (the high-temperature, pure metallic state) at a step of 0.2. Each spectrum is normalised against a plain VO_2 film with the corresponding *f*. The incident light is polarised along (**a**) the short-axis and (**b**) the long-axis of the nanorods.
